# The Use of Digital Technology for COVID-19 Detection and Response Management in Indonesia: Mixed Methods Study

**DOI:** 10.2196/41308

**Published:** 2023-02-28

**Authors:** Dewi Nur Aisyah, Alfiano Fawwaz Lokopessy, Maryan Naman, Haniena Diva, Logan Manikam, Wiku Adisasmito, Zisis Kozlakidis

**Affiliations:** 1 Indonesia One Health University Network Depok Indonesia; 2 Department of Epidemiology and Public Health Institute of Epidemiology and Health Care University College London London United Kingdom; 3 Faculty of Public Health Universitas Indonesia Depok Indonesia; 4 Aceso Global Health Consultants Pte Limited Singapore Singapore; 5 International Agency for Research on Cancer World Health Organization Lyon France

**Keywords:** COVID-19, Indonesia, digital technology, digital innovation, digital health, response management, robot innovation, decontamination

## Abstract

**Background:**

The COVID-19 pandemic has triggered a greater use of digital technologies as part of the health care response in many countries, including Indonesia. It is the world’s fourth-most populous nation and Southeast Asia’s most populous country, with considerable public health pressures.

**Objective:**

The aim of our study is to identify and review the use of digital health technologies in COVID-19 detection and response management in Indonesia.

**Methods:**

We conducted a literature review of publicly accessible information in technical and scientific journals, as well as news articles from September 2020 to August 2022 to identify the use case examples of digital technologies in COVID-19 detection and response management in Indonesia.

**Results:**

The results are presented in 3 groups, namely (1) big data, artificial intelligence, and machine learning (technologies for the collection or processing of data); (2) health care system technologies (acting at the public health level); and (3) COVID-19 screening, population treatment, and prevention population treatment (acting at the individual patient level). Some of these technologies are the result of government-academia-private sector collaborations during the pandemic, which represent a novel, multisectoral practice in Indonesia within the public health care ecosystem. A small number of the identified technologies pre-existed the pandemic but were upgraded and adapted for current needs.

**Conclusions:**

Digital technologies were developed in Indonesia during the pandemic, with a direct impact on supporting COVID-19 management, detection, response, and treatment. They addressed different areas of the technological spectrum and with different levels of adoption, ranging from local to regional to national. The indirect impact of this wave of technological creation and use is a strong foundation for fostering future multisectoral collaboration within the national health care system of Indonesia.

## Introduction

For the past 2 decades, Indonesia has faced various outbreaks of emerging and re-emerging infectious diseases, such as measles [1], SARS [2], MERS [3], H5N1 [4], H1N1 [5], and, most recently, COVID-19 [6]. These have challenged individuals, health care systems, and infrastructures on how to best prevent wider community transmission, how to treat patients effectively, and how to suppress cases until finally the disease outbreak can be controlled. The COVID-19 pandemic has created a global emergency that requires many different parties to collaborate in a coordinated and systematic manner to implement health policies and community actions. As with previous crises, they can act as a catalyst and trigger new ideas and innovations [7-9]. As a result, multiple breakthroughs and scaled-up implementations in digital technology have also emerged during this pandemic [10]. Some of them have contributed to the surveillance, detection, or responses of positive cases and their direct contacts [11,12], which helps policy makers manage the pandemic, including in Indonesia. Some are pre-existing technologies, such as remote consultations, whose use is being enhanced or modified for the pandemic [13-15].

Several recently published studies have presented the use of digital technologies during the COVID-19 pandemic in several countries. For example, Whitelaw et al [16] grouped technologies used in more than 10 countries into different functions for pandemic planning and responses, such as tracking, screening for infection, contact tracing, quarantine and self-isolation, and clinical management. Another study in Saudi Arabia also found the use of digital technologies at different stages of pandemic responses, namely at digital screening, surveillance, contact notification, and follow-up [17]. Although there might not exist an international consensus on the grouping of these technologies currently, these models remain useful in being able to navigate and study the field as well as the impact of implementing such technologies.

More specifically, in Indonesia, the use for many of these digital technologies has been promoted and enhanced by the government throughout the pandemic, through various innovation and research programs [18] and by the incorporation of digital technologies as part of routine data collection activities, supporting evidence-based policy making [19]. The surge in the number of COVID-19 cases and deaths has resulted in the tightening of barrier measures (eg, masks, personal protective equipment) and population movement restrictions (eg, curfew in large urban centers). These have placed a strategic focus on digital technology use in responding to the pandemic, such as through the recent government partnerships with telemedicine apps to provide free remote medical consultation for faster responses and easing potential crowding at hospitals [20]. During the pandemic, Indonesia, the most populous nation of Southeast Asia, experienced waves with corresponding sharp rises in cases and deaths (eg, 60% of positive cases increased in the week of June 19-29, 2021, alone [7] as compared to the immediately prior week). Altogether, Indonesia had recorded over 2.3 million cumulative cases and over 61,000 cumulative deaths as of July 5, 2021 [8], which led to government restrictions on people’s mobility in its most populous islands of Java and Bali from July 3 to 20, 2021 [10]. This imposition of population movement restrictions was repeated on several occasions throughout 2021 and 2022. Drawing from the use of digital technologies in other countries, as well as the authors’ expertise on the implementation of digital health technologies within Indonesia [21], this paper aims to identify and classify the use of digital technologies for COVID-19 detection and response management in Indonesia. By combining multiple sources in English and Indonesian, in scientific peer-reviewed literature, as well as information released by governmental departments, the authors believe that they can provide an exhaustive and detailed narrative review of the emerging digital health landscape.

## Methods

### Search Strategy and Selection Criteria

We considered any studies that reported the use of mobile apps or digital technologies or both that support COVID-19 pandemic control in Indonesia. We conducted a systematic literature search using OVID Embase, OVID Medline, and PubMed databases, as well as the Google Scholar search engine, using the terms “digital” or “technology*” or “robotic” or “tracing” or “dashboard” or “telemedicine” AND “COVID-19” or “coronavirus disease” or “SARS-Cov-2” AND “Indonesia.” To expand the literature search, we also conducted manual searches through publicly accessible regional and national official announcements, press releases, and published data within Indonesia. We included studies that were published in English after January 2020 (ie, from the time of the first confirmed positive SARS-CoV-2 case in Indonesia to the time of the authoring of this paper) up to August 2022.

The inclusion criteria were (1) usage/practice related to the COVID-19 pandemic detection and response in Indonesia and (2) the use of digital technology and digitization of services directly related to the pandemic needs. We excluded studies that were not relevant to COVID-19 pandemic control and response, as well as studies that were not implemented in Indonesia. There were no exclusions in relation to the type of technology creator or the type of technology user (eg, public, private, or mixed consortia). All identified manuscripts were reviewed independently by 2 authors, and all those that referred to or presented specific information in relation to the implementation of a digital health technology for COVID-19 in Indonesia were included. The resulting list was confirmed by independent review by a third author. The review process followed Preferred Reporting Items for Systematic Reviews and Meta-Analyses (PRISMA) guidelines.

### Thematic Validation

Due to the limited number of identified manuscripts and to reach thematic validation, the authors interviewed (noncompensated) additionally 10 individuals from key stakeholder organizations (ie, 2 individuals from each of the institutions involved in digital technology use for COVID-19, namely the Ministry of Health, the COVID-19 National Taskforce, Telkom Indonesia, the Indonesian Red Cross, and the DKI Jakarta Local Government). This allowed for independent thematic validation, as derived from the initial data collection round. Subsequently, the authors collected, studied, and organized all the information obtained to determine (1) key practices when digital technologies were used as well as (2) any lessons learned. Preliminary thematic groups were generated and were linked to interview texts using traditional content analysis. Emerging themes were discussed and presented at team meetings. During these meetings, discordant classifications were discussed until a consensus was reached.

### Ethics Statement

There was no patient involvement in this review. All interviews were conducted anonymously. Although participants were not signing a separate consent form, consent was obtained by completion of the interview. Thus, a waiver was obtained from the International Agency for Research on Cancer Ethics Committee (reference number 22-11362).

## Results

### Paper Screening

**Figure 1:**
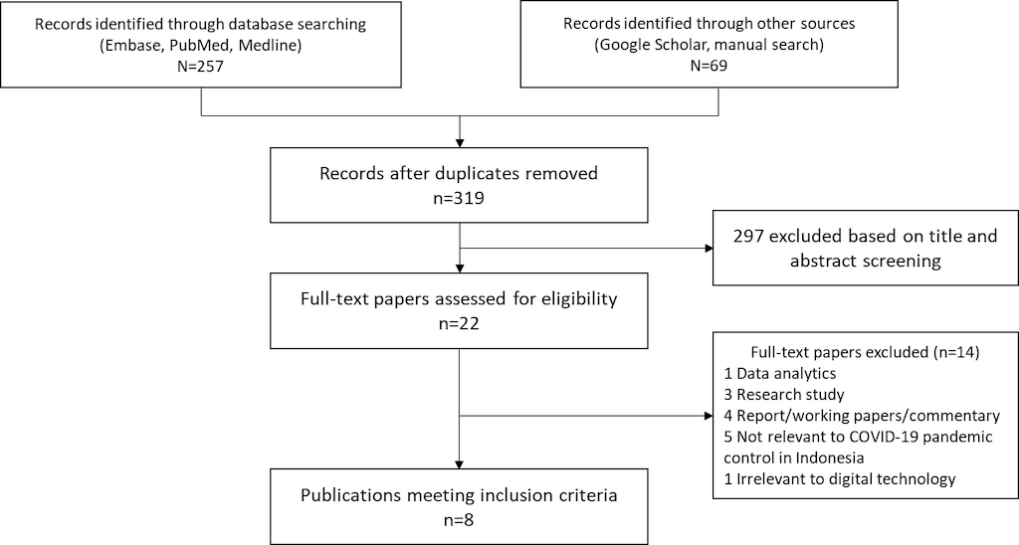
Paper screening following PRISMA guidelines. PRISMA: Preferred Reporting Items for Systematic Reviews and Meta-Analyses.

After excluding duplicates, we retrieved 319 papers from 3 databases and 1 from the gray literature. Most papers had simple mentions of the need to implement technological solutions within Indonesia, but few contained actual examples of doing so. Of the 319 papers, 22 (6.9%) met the criteria for full-text review. Finally, we identified 8 (36.4%) studies describing the use of digital technology to support the COVID-19 pandemic control and response in Indonesia (Figure 1). The list of included information and communications technology (ICT) tools is provided in Multimedia Appendix 1.

In Indonesia, as with many countries globally, a public health response and management system was built and implemented through COVID-19 detection, prevention, treatment, and monitoring. In total, 36 digital technologies were identified supporting the management aspects and 11 digital technologies identified supporting treatment, such as telemedicine apps providing free COVID-19 consultation and treatment for patients with mild symptoms and information for asymptomatic individuals. Each of the latter technologies facilitates a mix of telemedicine consultations and medicine delivery services for patients with COVID-19 [22]. Among the latter set of 11 technologies, 10 (90.9%) predated the pandemic, and their use was enhanced during the pandemic, while 1 (9.1%) was launched during the pandemic. Namely, those 11 digital apps are Alodok (PT Sumo Teknologi Solusi), GetWell (PT Telemedika Teknologi Indonesia), Good Doctor (Good Doctor Tech), GrabHealth (PT. Grab Indonesia), Halodoc (PT Media Dokter Investama), KlikDokter (PT Medika Komunika Teknologi), KlinikGo (PT Medika Nuswantara Digital), Link Sehat (PT Link Medis Sehat), Milvik Dokter (PT Milvik Indonesia), ProSehat (PT Atoma Medical), SehatQ (PT SehatQ Harsana Emedika), and YesDok (PT Yes Dok Indonesia).

Additionally, 9 robot innovation products were developed to support health workers treating hospitalized patients. Namely, the 9 robotics innovation products are Robot RAISA TIARA, Robot RAISA BCL, Robot Dekontaminasi (decontamination robot), Smart Syringe Pump, Autonomous UVC Mobile Robot, Robot Violeta, Smart Telemedicine Robot “Win-MTA,” Service Robot, and Doctor Representative Robot. Their intended aims are to prevent within-hospital transmission or to alleviate health care workers’ work burden. In addition, 2 health care data systems were developed assisting patients’ treatment, namely SIRANAP (Sistem Informasi Rawat Inap) and Blood Plasma Donor. These 2 data systems help individuals with COVID-19 to find available hospital beds and blood plasma.

It is worth noting that the number of such available apps has grown quickly since the pandemic started in Indonesia. Overall, 1 new telemedicine app, 1 new health care data system app, 5 new mobile phone–based apps, and 1 big data/machine learning analytics platform were developed and launched during the pandemic. These digital technologies have been developed almost entirely as multisectoral government-university-private sector partnerships.

**Figure 2:**
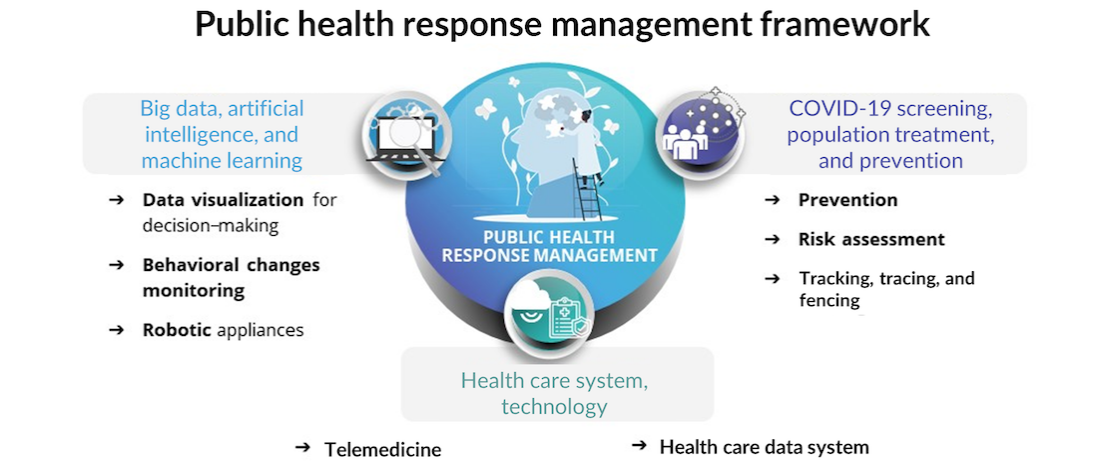
The use of digital technologies for detection and response management of the COVID-19 pandemic in Indonesia, divided into 3 main categories.

To study and classify these digital emerging technologies in a more systematic way, they were classified into 3 major user groups, as illustrated in Figure 2, namely (1) big data, artificial intelligence, and machine learning (ie, technologies for the collection, integration, ingestion, and processing of data, as well as robotic systems); (2) health care system technologies (ie, technologies acting at the individual level), and (3) COVID-19 screening, population treatment, and prevention (ie, technologies acting at the population level). These 3 major groups are further expanded next.

### Big Data, Artificial Intelligence, and Machine Learning

Three types of uses were identified within this theme: (1) data visualization, (2) behavioral change monitoring, and (3) robotic appliances. Although the first 2 have primarily contributed to evidence-based policy making, the third supported health workers in treating patients.

In terms of data collation and visualization tools for decision-making support, Indonesia has 34 provinces that comprise districts and cities with decentralized local governments. Of these, 7 (20.6%) provinces developed websites and dashboards independently and customized them to their needs and local context to aggregate and manage COVID-19–related data, to keep the public and policy makers updated with the current situation, and to fact-check available information and provide relevant contact numbers for seeking medical services and treatment. Table 1 shows some examples of those websites and their respective dashboards.

**Figure 3:**
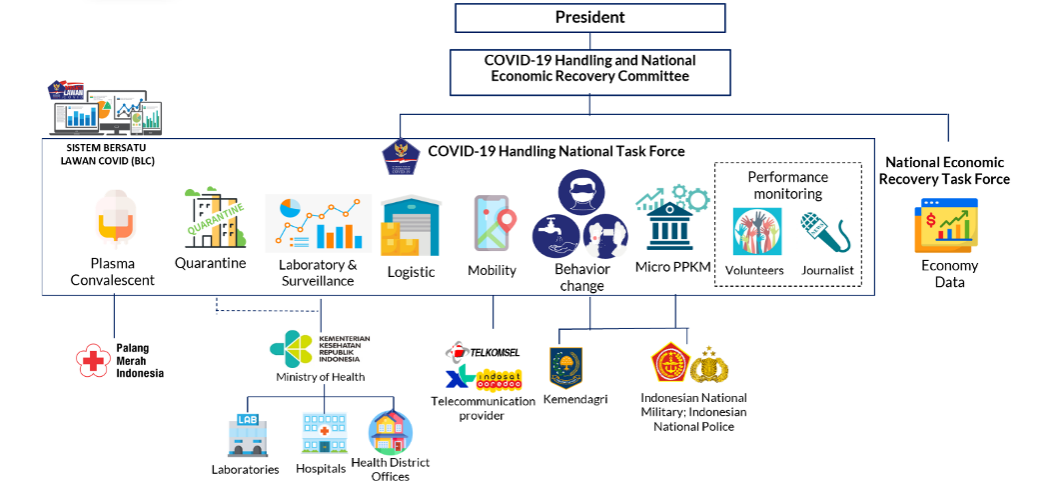
Functions and interoperability of BLC national data. BLC: Bersatu Lawan COVID-19; PPKM; Pemberlakuan Pembatasan Kegiatan Masyarakat (Community Mobility Restriction).

For health protocol compliance/behavioral change monitoring, health protocol compliance–monitoring systems were all analyzed using the interoperable platform *Bersatu Lawan COVID-19 (BLC)*, which translates as “United Against COVID-19,” that any government level can access, be it national, provincial, or district/city government. This system uses big data analysis that allows real-time monitoring of compliance to health protocols, such as mask wearing and keeping social distance, to inform policy makers on public behavioral changes [23]. Figure 3 depicts its function and interoperability with other COVID-19–related data supplied to Indonesian policy makers.

**Figure 4:**
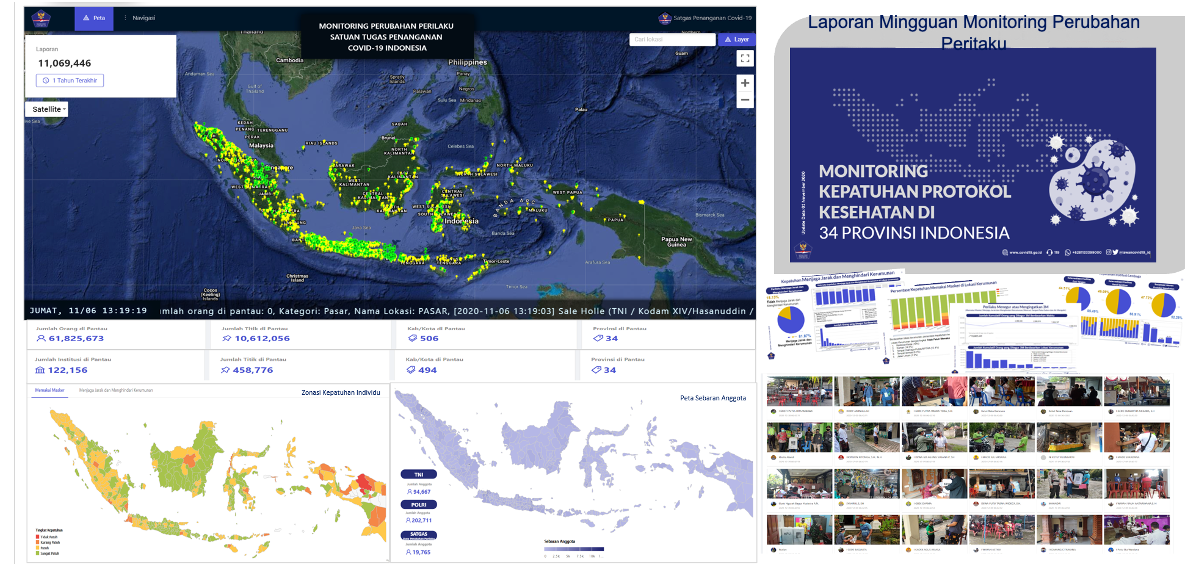
Dashboard for the Health Protocol Compliance Monitoring System.

One of the flagship products of the BLC is the Health Protocol Compliance Monitoring System that has been supporting policy makers nationwide in observing the compliance of key public spaces. Through this data system, the Indonesian military (TNI), police (POLRI) personnel, and volunteer community ambassadors can submit reports from key public spaces, such as markets and train stations, on whether people have complied with wearing face masks and keeping a social distance of minimum 1 m. Figure 4 shows the dashboard and resulting reports created from these analyses made publicly accessible [24]. Since its launch in October 2020 and until December 2021, the BLC app has facilitated more than 211.3 million health protocol compliance–monitoring reports, with 721.4 million people monitored and 165,537,934 locations under observation in all of the 514 districts/cities in the 34 provinces in Indonesia.

In regard to robotic appliances, as the COVID-19 pandemic necessitated limited physical contact between health workers and patients, innovations emerged in robotic technologies by various government and higher education institutions assisting in the treating of patients with COVID-19 in various hospitals and institutions [17]. A study in China found a similar increase in the use of robotics to minimize physical contact and also found that robotics can help reduce the risks of health care workers getting infected by COVID-19 [25]. Additionally, robotic technologies may help in processing information, delivering food or medicine, carrying out temperature checks, and carrying out disinfection tasks. Table 2 compiles a list of the robotic innovations introduced in Indonesia in direct relation to clinical COVID-19 management [17].

**Table 1:** List of several COVID-19–related data collation and visualization tools.

Website or dashboard name	Functions
Pikobar (Pusat Informasi & Koordinasi COVID-19 di Provinsi Jawa Barat)	Presenting statistical updates on new positive cases, self-isolating people, hospitalization, suspected cases, probabilities, and contact tracing
Executive Information System Dinkes Provinsi DKI Jakarta	Presenting real-time information about isolation bed availability
Jakarta Tanggap COVID-19	Presenting information about COVID-19 cases in Jakarta
Pusat Informasi & Koordinasi Kota Depok Jawa Barat	Presenting information about COVID-19 cases in Depok and providing hotline services
COVID-19 NTB (Nusa Tenggara Barat)	Presenting information about COVID-19 cases in Depok and providing hotline services and fact-checking service
Sulsel Tanggap COVID-19	Presenting information about COVID-19 cases in Depok and providing hotline services and fact-checking service
Pusat Informasi COVID-19 Provinsi Maluku	Presenting information about COVID-19 cases in Depok and providing hotline services and fact-checking service

**Table 2:** List of robot innovation products related to COVID-19 management in Indonesia.

Robot name	Innovator	Functions	Other remarks	Status
Robot RAISA	Institut Teknologi Sepuluh Nopember Surabaya and Universitas Airlangga	Providing medical assistant and nurse-like functions	The robot can carry up to 5 kg of weight, has a camera, and can facilitate 2-way communication between health workers and patients.	Used in the University of Airlangga Hospital
Robot RAISA TIARA, Robot RAISA BCL^a^	Institut Teknologi Sepuluh Nopember Surabaya and Universitas Airlangga	Opening ICU^b^ doors and checking the patient’s body temperature, oxygen saturation, and heartbeat.	The robot can also remotely observe infusion drops and urine production from up to a 5 km distance and includes a 360° rotation function.	Prototype produced and used in at least 6 hospitals: Universitas Airlangga (UNAIR) Hospital, Dr Soetomo Hospital, Husada Utama Surabaya Hospital, Saiful Anwar Malang Hospital, Wisma Atlit, RSI Surabaya
Robot Dekontaminasi	Institut Teknologi Sepuluh Nopember Surabaya and Universitas Airlangga	Decontaminating used items and used personal protective equipment (PPE)	The robot is operated using a remote control.	Prototype produced and ready to be used
Smart Syringe Pump	Institut Teknologi Sepuluh Nopember Surabaya and Universitas Airlangga	Automatically filling medicine administered to the patient with a set schedule	A mobile app remotely operates the pump.	Prototype produced and ready to be used
Autonomous UVC Mobile Robot	Telkom University and Indonesia Institute of Sciences (LIPI)	Disinfecting and sterilizing isolation rooms for patients with COVID-19	The robot is equipped with UVC light to effectively kill coronavirus.	Prototype produced and tested and has been used in hospitals in West Java Province
Robot Violeta	Institut Teknologi Sepuluh Nopember Surabaya and Universitas Airlangga	Eliminating or decelerating the growth of pathogens by using UV 200-300 nm waves	Using remotely operated UV light from a 1-2 m distance, the robot takes 10-15 minutes to perform the sterilization task.	Used in the University of Airlangga Hospital
Smart Telemedicine Robot “Win-MTA”	Universitas Gadjah Mada and PT. Maetala Visionaire Tecnologia	Disinfecting objects using >39°C temperature, delivering medicine and prescriptions to patients, and sending patients’ data (temperature, GPS location)	N/A^c^	Remains a prototype
Robot Pelayan	Universitas Gadjah Mada and Academic Hospital, Universitas Gadjah Mada	Automatically locating the patient’s room and providing automated medicine and food delivery services to rooms	N/A	Remains a prototype
Doctor Representative Robot (Doper)	Telkom University	Facilitating medical and nutrition consultation without physical contact with health workers	N/A	Under development

^a^BLC: Bersatu Lawan COVID-19.

^b^ICU: intensive care unit.

^c^N/A: not applicable.

### Health Care System Technology

Three types of uses were identified within this theme, namely telemedicine, digital support for quarantine, and health care data systems. All of them were developed with the aim to provide services for those self-isolating, seeking medical treatment, or seeking hospitalization.

#### Telemedicine and Quarantine Digital Support

Telemedicine is an example of digital technology already developed and used for health purposes before the pandemic within Indonesia, albeit sporadically. The World Health Organization (WHO) already discussed the potential benefits of telemedicine in overcoming distance barriers and speeding up health care delivery, while considering current high technological costs [26]. Portnoy et al [27] observed a rising use of telemedicine in the 2-3 years preceding the pandemic, and further growth during the pandemic, as quick medical advice could be provided to patients with mild symptoms, thus allowing for timely, accurate information dissemination and indirectly limiting community transmission. Further examples include apps allowing health care workers who are self-isolating to continue supporting patients remotely [28]. Moreover, research in China found this approach to be an effective solution to minimize infection risks to health workers by minimizing physical contact [29].

Despite these promising benefits, challenges, such as high cost [26] and integration with extant national health care systems, persist. In Indonesia, the surge in COVID-19 cases and the tighter population movement restrictions in July 202 brought to the fore a partnership between the government and 11 telemedicine mobile apps [22]. This umbrella partnership enabled the government to cover the cost of services and allowed patients to access medical advice and medications for free. Thus, the Indonesian government responded to the public health pressures rising from the surge in COVID-19–positive cases, while temporarily overcoming the cost challenge that is faced by telemedicine apps in other countries. It also allowed for limited integration with the national COVID-19 surveillance mechanism by providing access to COVID-19 testing. Table 3 compiles a list of the telemedicine mobile app partners of the Ministry of Health of Indonesia.

**Table 3:** List of telemedicine mobile app partners of the Ministry of Health.

Telemedicine mobile app	Year of launch	Number of users	Features
Alodokter	2014	40 million	Chat with doctors, health articles, consultation booking with doctors, online drug store, Alodokter insurance
GetWell	2021	>5000	Chat and video calls with doctors, personal health records, 24/7 panic button, health articles, integrated with PeduliLindungi app (government’s COVID-19 and vaccination status app)
Good Doctor	2020	>1 million	Consultation with doctors, online drug store and delivery, doctor appointment-booking system, insurance claim
Halodoc	2016	20 million (2021)	Chat and video calls with doctors, see the doctor’s experience and rating, a health store, booking a hospital doctor, getting a laboratory test, linking insurance
KlikDokter	2015	>1 million	Live chat with doctors, hospital appointment booking, pregnancy journey tracker (via the HalloBumil app), period tracker and calendar, heart and diabetes risk measurement, BMI calculator
KlinikGo	2022	>10	Online booking for hospital appointment
Link Sehat	2020	>10,000	Consultation with doctors, COVID-19–testing appointment, hospital schedule booking, online medical assistance, health articles
Milvik Dokter	2019	>10,000	Consultation with doctors, medicine and laboratory check
ProSehat	2015	>100,000	Online chat with doctors, home visits, getting a laboratory test, drive-through immunization, product delivery
SehatQ	2019	>500,000	Chat with doctors, buy drugs, pregnancy consultation and discussion forum, mental health, and other services
YesDok	2017	>100,000	Consultation with doctors (prediagnosis, first aid, education, medicine recommendation, and consultation playback), drug delivery

In addition to the telemedicine mobile apps partners of the Ministry of Health, many hospitals also provided telemedicine service using mobile apps, including the Cipto Mangunkusumo Hospital with the SiapDok app, the Siloam Hospital Group with the AIDO app, and the YARSI Hospital with the MAUDOK app. These telemedicine apps were organization specific, with the aim to help hospital patients arrange online appointments as well as receive health consultations with doctors.

The 2 most popular telemedicine apps in Indonesia are Halodoc, with monthly active users reaching 20 million [30], followed by Alodokter, with monthly active users being around 18 million [31]. According to another survey conducted by the Katadata Insight Center, during the COVID-19 pandemic, the most popular telemedicine apps were Halodoc (46.5%), followed by telemedicine provided by hospitals (41.7%) and Alodokter (35.7%) [32].

In principle, the features of teleconsultation with doctors in these apps are similar. The differentiating features are the user interface (UI) and user experience (UX) aspects, as well as the promotion(s) offered by the operating company, such as medicine delivery and cashback offers.

#### Health Care Data System (Hospital, Laboratory, and Tracing)

The surging cases and the rising needs for hospitalization have created unprecedented bed occupancy rates in hospitals in Indonesia, leading to difficulties in finding available beds for patients, especially those requiring urgent attention. The more than 12,000 new COVID-19–positive cases and more than 175,000 active cases recorded at the end of January 2021 made the government launch an additional health care system to check hospital bed availability, especially intensive care units (ICUs) in all 34 provinces [33]. By July 2021, as Indonesia faced a subsequent wave with a sharp rise in the number of cases and hospitalizations, an additional health care data system was launched for individuals in need of real-time access to information, such as access to oxygen [34] and to receive and donate convalescent plasma [35].

In addition to supporting COVID-19 case identification and laboratory test result integration across Indonesia, the Ministry of Health developed New All Records (NAR) TC-19 for all COVID-19 laboratory networks to input both polymerase chain reaction (PCR) and antigen test results. At the beginning of the pandemic, only the National Institute of Health and Research Development (NIHRD) had the capacity for COVID-19 testing [36]. Through extended collaboration with other ministries, institutions, nongovernmental organizations (NGOs), international donors, and the private sector, the number of COVID-19 laboratory networks that were using NAR increased to 936 across all 34 of Indonesia's provinces as of June 2022 [37]. SILACAK was also developed by the Ministry of Health to strengthen contact-tracing efforts in Indonesia [38]. Table 4 contains the 4 health care data systems, including hospital, laboratory, and contact-tracing data systems.

**Table 4:** List of health care and laboratory data systems.

Website or dashboard	Functions
SIRANAP^a^	The SIRANAP platform provides beds and ICU^b^ availability data. Hospitals provide real-time updates through this platform every 3 hours, recently with additional volunteer support from IndoRelawan. This feature has already merged into the PeduliLindungi app.
Blood Plasma Donor	The system provided access to people who have recovered from COVID-19 and are eligible for convalescent plasma donation.
NAR^c^ TC-19	Laboratory test data results are integrated into the system both for PCR^d^ and the antigen test.
SILACAK	Data collection tools for health care workers are used to carry out contact tracing in the community.

^a^SIRANAP: Sistem Informasi Rawat Inap (translates as “hospitalization information system”).

^b^ICU: intensive care unit.

^c^NAR: New All Records.

^d^PCR: polymerase chain reaction.

### COVID-19 Screening and Population Treatment

This last group of findings compiles the use of digital technologies for prevention, risk assessment, and contact tracing. These 3 functions are often found combined within a single platform. The following list details the platforms offering these:

- PeduliLindungi, which means “to care for and to protect,” is a smartphone-based app released by the Ministry of Communication and Information Technology of the Republic of Indonesia that the public can use for self-assessment, for example, to know the COVID-19 infection risk within their surroundings using the government's population and contact-tracing databases [39,40]. Additionally, the app is also synchronized with vaccination data, where people can check whether they are eligible for vaccination and register to receive one. The app also provides a list of nearby vaccine centers. People who have been fully vaccinated can also access their vaccine certificates through the app [41].
- Corona Likelihood Metric (CLM) is a COVID-19–screening mobile app that provides an online self-assessment form with the help of machine learning technology formulated by the Government of DKI Jakarta Province, in collaboration with the Harvard CLM Team and Klakklik. CLM can also recommend whether a citizen should take a COVID-19 test [39].
- Fight Covid-19 is a mobile app used by 1 of the local governments, namely Bangka Belitung Province, to trace the mobility of people from COVID-19 epicenter provinces, such as Jakarta to Bangka Belitung. The app is used to track the travel history of arrivals using location data collected from the phone GPS [40].
- Blue Pass is a device for contact tracing within an office setting. This device has been successfully used in Singapore, and a trial took place at the National Disaster Mitigation Body (BNPB), Indonesia. The device uses the GPS to record people who stand within a 3 m distance from another person. When one person tests positive for COVID-19, all recorded people who ever stood within the 3 m radius are notified as a form of contact tracing [42].
- Electronic Health Alert Card (e-HAC) is a mobile app being used to record people’s international mobility to Indonesia and people’s mobility within Indonesian provinces. All passengers of airplanes, ships, and trains are obliged to fill in their travel record data (destination and origin) to be able to enter the Indonesian border and travel domestically. The app is also connected with official clinics in Indonesia, where people can get COVID-19 tests before traveling; thus, it can record COVID-19 test results for domestic travel [43].
- 10 Rumah Aman is a mobile app developed by the Kantor Staf Presiden (KSP, or President Staff Office) and the Kementerian Komunikasi dan Informatika (Kemenkominfo, or the Ministry of Communication and Information Technology) to educate the community about the COVID-19 pandemic and recommend preventative actions, such as routine temperature checking. Several features are displayed in the app, such as “Check Body Temperature,” “Become a Safe Warrior,” “Information for Your Health,” “Regarding COVID-19,” “Check Your Health Here,” “COVID-19 WhatsApp,” and “Monitor Body Temperature Map.”
- Provincial mobile apps (Pusat Informasi dan Koordinasi COVID-19 Jawa Barat [PIKOBAR], Sawarna, Pantau Pandemi Sulawesi Barat [Papa Sulbar]): A special mention should be made for some provinces that also created or adopted COVID-19 features on their mobile apps specifically for their local populations. For example, PIKOBAR (or the West Java COVID-19 Information and Coordination Center) provides information about COVID-19 case distribution across West Java, information about the schedule and location for COVID-19 vaccinations, information about self-quarantine, COVID-19 logistic requests, and hospital and call center contact numbers. In Bandung City, the local government created Sawarna, a mobile app that helps the local community know the COVID-19 case distribution in Bandung. Another mobile app available is Papa Sulbar (or the Pandemic Monitor at West Sulawesi) that provides information about COVID-19 case distribution in West Sulawesi as well as the latest updates of COVID-19 pandemic control across the province.
- Mobile JKN is a mobile app developed by the Badan Penyelenggara Jaminan Sosial (BPJS, or the Social Health Insurance Administration Body) to facilitate access for BPJS participants based on a prepandemic beta version, which was subsequently further enhanced. Using Mobile JKN, individuals can get information about BPJS, such as checking membership, paying dues, checking health facilities, and requesting reprinting of membership cards. During the COVID-19 pandemic, mobile JKN also adopted a COVID-19 self-screening feature.

Several countries have adopted population screening apps to aid in the control of the pandemic waves as well as to function as a reference and warning point for individual users (eg, if the latter were colocated in time and space with known individuals with COVID-19). Such examples include the Corona Warn app in Germany [44], the CovidSafe app in Australia [45], and Covid Tracker in Ireland [46]. However, the adoption rates of such apps were altogether lower than originally anticipated (eg, the government-endorsed CovidSafe app in Australia was installed by 21.6% of the population, and that is 1 of the highest adoption rates observed), primarily due to concerns about personal data security.

The data visualization tools described in this study still exist and are being used to provide updated information about COVID-19 cases at national and local levels. The Health Protocol Compliance Monitoring System is currently still being used; however, the number of reports has decreased. Telemedicine apps are still being used, and health care data systems, such as SIRANAP, have been integrated into PeduliLindungi. Blood Plasma Donor is no longer active since WHO did not recommend blood plasma convalescent transfers for patients with COVID-19 since December 2021 [47]. Regarding COVID-19 population screening and treatment, (1) PeduliLindungi has been downloaded by more than 90 million of the Indonesian population and will become a citizen's health app adopted by the Ministry of Health [48]; (2) the CLM is still available on the DKI Jakarta local government website, although the usage is low; (3) Fight Covid is still used in Bangka Belitung Province, although the usage has decreased; (4) Blue Pass has been implemented at several places, such as the BNPB, Bintan resorts, and other resorts or tourist attractions. However, Blue Pass is no longer widely used compared to the first launch at the beginning of 2021; and (5) e-HAC is no longer used, although its function has been integrated into PeduliLindungi.

## Discussion

### Principal Findings

History has shown that major crises can often trigger new ideas and innovations [49]. In this context, the digital technologies that have been developed for COVID-19 in Indonesia and were identified in this review represent a leap forward for Indonesian digital health innovation. The pandemic has afforded the opportunity for the largest number of health technologies ever (almost 50 different technologies in total) to be introduced into the Indonesian health care ecosystem.

As a developing country, Indonesia can learn more from other developed countries in Asia, such as South Korea using the MERS-CoV outbreak in 2015 as its turning point to advance digital health use and innovation within its health care services [23]. Our findings demonstrate an opportunity for these technologies to impact many different areas of the Indonesian health care services, as the digital health technologies introduced cover a wide number of applications (from decision-making support and encompassing health system technologies to robotics to individual patient monitoring and tracing). Several international studies have also highlighted the need for developing countries to accelerate digital innovation, given the gaps in research and innovation, in digital health [49,50] particularly in promoting learning systems that foster ongoing collaborations between government, industry (private sector), academia, and community, sometimes called the “quadruple helix of innovation” [51]. There have been several efforts from global health organizations, such as the WHO Access to COVID-19 Tools (ACT) Accelerator Diagnostics Partnership. This initiative focused on bringing high-quality rapid tests, training professionals, and establishing testing for over 500 million people in low- and middle-income countries [52]. As this review demonstrates, the majority of health care technologies introduced were multisectoral with a wide potential reach, with the exception of 3 local provincial apps and 1 institutional one (BPJS) that were more limited in their offerings. Thus, the pandemic has created a precedent for further multisectoral development of health care innovation with a potential national rollout.

However, this work also identified some of the challenges Indonesia is facing to advance the use and innovation of digital technologies as follows: First, the data privacy challenge emerged alongside the invention and use of many digital technologies. This is not an exclusive issue to Indonesia, as it has previously been reported regionally for South Korea, Singapore, Taiwan, and Hong Kong [23]. South Korea and Taiwan have been using electronic wristbands to prevent people from violating self-isolation by using location-tracking systems. In Singapore, TraceTogether is dedicated as a contact-tracing platform using Bluetooth, while in Taiwan, a similar platform uses a digital fencing system. The usage of these tracking or fencing tools raises questions regarding the protection of privacy. However, the Singaporean government has anticipated debates toward the importance of privacy and data protection [53]. The system will not store data, not even geolocation data, and the users’ phone numbers and personal identification data are not exchanged at any point. In Indonesia, at an individual user level, the government has protected the users’ privacy by not storing geolocation data in the local app, in addition to not exchanging any user information and disallowing permission options to access users’ data [54]. At a population level, a further concern relates to the analysis and publication of the gathered data, since COVID-19 infections are being publicly reported extensively, potentially risking the leak of patients’ personal data [23]. In response to this issue, Indonesia is improving its information technology regulation to ensure users’ safety and privacy [25]. Government-led apps, such as PeduliLindungi and others have set an example and updated their user agreement and privacy policies in parallel and in line with the governmental initiative to ensure users’ trust in using the apps safely [41]. To protect data and privacy related to COVID-19, the Ministry of Health worked together with the National Cyber and Crypto Agency (BSSN), an agency under the Government of Indonesia with relevant expertise. Thus, although the existing data safety and privacy solutions might not be in their final form and might still require further updating in the future, the implemented solutions so far are functional.

Second, several implementation challenges remain prevalent in Indonesia’s use of digital technologies. These include (1) users’ adoption of mobile apps, (2) digital literacy and disparities of technology use among provinces, (3) data analysis that is often hampered by multisectoral coordination, and (4) the need to invest in human resources to foster innovation in the mid- and longer term. The initial low user adoption for apps triggered the government’s issuance of regulation to mandatorily use specific apps, such as in the case of e-HAC [55]. In doing so, the debate enhanced relating to data privacy, data collection, and how much an app provider or a local or national government should know about its citizens.

Regarding the second challenge identified, technological disparity (as in a combined effect of digital literacy and disparity of access to new technologies) was existing prepandemic and perhaps has been consolidated further during the COVID-19 pandemic, with certain population groups benefiting from multiple digital health technologies (eg, in the capital Jakarta), while rural populations have fewer options available to them. This aspect was mentioned in all but 1 of the manuscripts considered in this paper where innovations are mainly promoted by major provinces in Java and the western part of Indonesia, exposing years of inequalities in education and wealth between the islands in the world’s largest archipelagic state [56]. Finally, regarding challenges (3) and (4), these have been major persistent issues prepandemic and these have come to the fore during the COVID-19 handling in Indonesia [57], with the country still working to develop frameworks that will foster multisectoral coordination for optimum data sharing and analysis. It is likely to remain high in the policy-making agenda, as the evidence from the pandemic demonstrated the usefulness of data sharing, informing policy makers across different ministerial bodies, at national and subnational levels. Lastly, a need to upskill human resources (eg, develop digital literacy of front-line health care staff) will not only foster the current use of digital technologies but also become an important step in maintaining digital innovation beyond the duration of the pandemic. This outlook aligns with objective 4, point 78 on the WHO Global Strategy on Human Resources for Health Workforce 2030, which is to strengthen human resources for health information systems and build the human capital required to operate them [58].

### Strengths and Limitations

The strength of this study is that it used a systematic approach based on PRISMA guidelines to perform an extensive literature search. The study is the first digital health care technology investigation for Indonesia and will likely set a precedent for similar future investigations. Furthermore, we identified and included the gray literature to ensure that as many as possible digital apps were captured.

However, there are also limitations to this study. Despite the intensive literature search, only a limited number of papers discussing digital technology implementation in Indonesia were identified. In particular, robotic technologies were much less mature than mobile-based platforms, and as such, their relative level of readiness (and overall impact) was difficult to estimate. Furthermore, most of the available information was commonly available in Indonesian languages, thus limiting the direct comparability of the information to other apps internationally. Finally, the relative percentage of the installation of these apps over the entire population of Indonesia is only known for few of the apps, and thus we were unable to provide a complete picture regarding the level of usage, frequency of usage, and overall level of reliance.

### Conclusion

This review presented the use of digital health technologies for COVID-19 detection and response management in Indonesia. The results were grouped into 3 broad use types for ease of analysis, namely (1) big data, artificial intelligence, and machine learning; (2) health care system technologies; and (3) COVID-19 screening, population treatment, and prevention. The introduction of these digital technologies represents the single-largest introduction of digital technologies within the Indonesian health care ecosystem. Additionally, almost all the technologies were the result of multisectoral coordination among government bodies at a national and subnational scale, along with higher education institutions, research institutions, and the private sector. Thus, the case of Indonesia may provide a blueprint for the introduction of digital health care technologies for several other resource-restricted settings. The introduction of these technologies demonstrated the potential benefit of big data for informing public health policy making during health emergencies, as initiated and led by the National Task Force for COVID-19 Mitigation Acceleration.

This work also acknowledges the many challenges that remain, such as data privacy, disparity of technological access, the need for further multisectoral coordination, and the ability to support such innovation by appropriately skilled staff. Therefore, although it is important to identify the benefits of the implemented digital health technologies, it also remains critical to maintain the multisectoral cooperation frameworks (among government bodies, academia, and the private sector) for the longer term, both for addressing population health needs and for advancing Indonesia’s digital health care transformation.

## Appendix

Multimedia Appendix 1 Preferred Reporting Items for Systematic Reviews and Meta-Analyses (PRISMA) checklist.

## Abbreviations

BLC: Bersatu Lawan COVID-19
BNPB: National Disaster Mitigation Body
BPJS: Badan Penyelenggara Jaminan Sosial
CLM: Corona Likelihood Metric
e-HAC: Electronic Health Alert Card
ICU: intensive care unit
NAR: New All Records
Papa Sulbar: Pantau Pandemi Sulawesi Barat
PCR: polymerase chain reaction
PIKOBAR: Pusat Informasi dan Koordinasi COVID-19 Jawa Barat
PRISMA: Preferred Reporting Items for Systematic Reviews and Meta-Analyses
SIRANAP: Sistem Informasi Rawat Inap
WHO: World Health Organization
